# Muscle Metabolism and Performance During Simulated Peak‐Intensity Periods Occurring Early and Late in a Soccer‐Specific Exercise Protocol in Well‐Trained Male Players

**DOI:** 10.1111/sms.70075

**Published:** 2025-05-15

**Authors:** Jeppe F. Vigh‐Larsen, Niels Ørtenblad, Victor Stoltz, Dan Fransson, Farzad Yousefian, Jeppe Panduro, Morten B. Randers, Thomas S. Ehlers, Peter Krustrup, Magni Mohr

**Affiliations:** ^1^ Department of Sports Science and Clinical Biomechanics, Faculty of Health Sciences University of Southern Denmark Odense Denmark; ^2^ Department of Movement and Sports Sciences, Faculty of Medicine and Health Sciences Ghent University Ghent Belgium; ^3^ Center for Health and Performance, Department of Food, Nutrition and Sport Science University of Gothenburg Gothenburg Sweden; ^4^ Research Centre in Sports Sciences, Health Sciences and Human Development (CIDESD), Department of Sport Sciences University of Beira Interior Covilhã Portugal; ^5^ Portugal Football School Portuguese Football Federation Oeiras Portugal; ^6^ Department of Nutrition, Exercise and Sports University of Copenhagen Copenhagen Denmark; ^7^ Danish Institute for Advanced Study (DIAS) University of Southern Denmark Odense Denmark; ^8^ Center of Health Science, Faculty of Health University of the Faroe Islands Tórshavn Faroe Islands

**Keywords:** energetics, fatigue, football, fuel, glycogen, metabolites

## Abstract

We applied a novel model mimicking the most intense 5‐min game periods to investigate muscle metabolic and fatigue responses to peak‐intensity exercise occurring early and late in a simulated soccer game. Eleven well‐trained male players completed a modified simulated soccer game (the Copenhagen Soccer Test) with 5‐min peak‐intensity period simulations inserted early (PP1; 10–15 min) and late (PP2; 85–90 min) in the game. Muscle biopsies and blood samples were obtained before and after each peak period. Muscle glycogen decreased during both peak periods (*p* < 0.001) by 62 ± 46 mmol kg^−1^ dw in PP1 and by 25 ± 37 mmol kg^−1^ dw in PP2, without a statistically significant difference in the glycogen breakdown in PP1 vs. PP2, despite a numerical trend (*p* = 0.115). Muscle lactate increased during both peak periods (*p* < 0.001) to 47 ± 25 mmol kg^−1^ dw and 32 ± 12 mmol kg^−1^ dw, with no clear difference in the increase (*p* = 0.108), despite blood lactate levels rising more in PP1 vs. PP2 (*p* = 0.031), reaching higher post PP1 levels (13.9 ± 3.6 mmol L^−1^ vs. 9.8 ± 2.4 mmol L^−1^, *p* = 0.003). Muscle ATP decreased by 4% (*p* = 0.004) and phosphocreatine by ~50% (*p* < 0.001) following both peak periods. RPE was higher during PP2 (10.0 ± 0.0 AU vs. 9.2 ± 0.8 AU, *p* = 0.023), while 10‐m sprint performance declined by ~10% (*p* < 0.001), with no differences between PP1 and PP2 (*p* = 0.280). In conclusion, a 5‐min peak period occurring early in a simulated game elicited a high anaerobic energy turnover, with marked muscle glycogen reductions, lactate accumulation, and PCr depletion. While high‐energy phosphate metabolism remained similar during the late peak period, glycogenolytic rate appeared attenuated, accompanied by aggravated perceived exertion but similar sprint performance deteriorations.

## 1 Introduction

Soccer is an intermittent team sport characterized by brief high‐intensity intervals, interspersed by longer duration low‐intensity sequences, repeated over the course of a game. This activity profile induces average heart rates of ~85%HR_max_, corresponding to ~70%VO_2max_ with surges toward maximal heart rates during the most intense periods of a game, which further comprises a proposed high anaerobic energy turnover [1]. Two decades ago, Mohr et al. [2] introduced the concept “temporary fatigue” within the context of a soccer game, which refers to transient impairments in exercise tolerance in relation to such peak‐intensity periods, often referred to as “peak periods”, encapsulating the most challenging 1–5 min game intervals endured defined based on various running intensity metrics [3, 4, 5]. Now, it is well established that elite soccer players perform less highintensity running immediately following peak periods, indicative of fatigue development; however, the acute physiological muscle metabolic and fatigue responses have not yet been objectively assessed [2, 3, 6, 7].

Muscle metabolism and fatigue development during single or repeated bouts of intense exercise have been studied in detail in laboratory settings commonly using cycle ergometry concomitant with sampling of muscle biopsies [8, 9, 10, 11, 12]. In such experiments, the energy turnover during a single 6‐s sprint, which could reflect a common intense soccer action, is nearly equally distributed between phosphocreatine (PCr) degradation and glycogenolysis, with an additional small contribution from oxidative phosphorylation (~10%) [8, 13]. However, during repeated bouts of high‐intensity exercise, muscle metabolism differs markedly, with a decrease in the rate of glycolysis partly compensated for by an increased oxidative phosphorylation [8, 13, 14]. Such repeated intense exercise leads to rapid glycogen breakdown and accompanying metabolic perturbations, including significant lactate accumulation and exhaustion of the PCr stores concomitant with decreased exercise tolerance [8, 11, 15].

In ecological settings of higher relevance to soccer, only two previous investigations have attempted to elucidate the muscle physiological responses to actual intense soccer match‐play sequences [16, 17]. Krustrup et al. obtained muscle biopsies in close proximity to intense periods during training games involving sub‐elite male players [16] and elite female players [17], reporting moderately elevated muscle lactate levels and only slightly reduced PCr concentrations, suggestive of a limited anaerobic contribution to the energy yield. However, in these studies, players were selected for muscle sampling in a random order after a period of intense actions, based on subjective evaluations, which is a limitation due to the large inter‐and intra‐game variability of these activities in soccer [18]. Furthermore, in these studies, there was a relatively long delay before muscle sampling (up to ~30 s) as players had to leave the pitch, while the male study included sub‐elite players who may exhibit different metabolic responses compared to elite players [16, 17]. As such, the acute muscle physiological and fatigue responses to peak‐intensity periods in soccer are not well examined.

To mitigate the impact of in‐game variability and establish a more standardized experimental framework, the use of simulated soccer models may provide a useful experimental approach. One such model is the Copenhagen Soccer Test (CST) [19], which simulates a soccer game and has been shown to provide a comparable physical loading akin to that of competitive play, encompassing similar cardiovascular and muscle metabolic responses. However, this model does not include peak periods where a substantial part of the high‐intensity work in a game is performed [6, 7]. Therefore, the inclusion of a peak period simulation could present a novel approach to address the muscle physiological responses to such intense game scenarios.

Importantly, peak periods can occur both early and late in a game, although the majority evidently occur in the initial stages, potentially attributed to subsequent fatigue development [1]. As such, several studies have demonstrated that fatigue accumulates throughout a soccer game [1], which may relate to several factors, including potentially altered muscle metabolism in association with partial depletion of the muscle glycogen levels [16, 17, 20, 21]. Indeed, the best muscle predictor of performance during the last 15‐min of a male soccer game has been shown to be the maximal activity of key regulatory enzymes in muscle β‐oxidation, which suggests that the ability to rely more on fatty acid oxidation and potentially spare muscle glycogen may be important [22]. Moreover, the availability and utilization of circulating substrates may be altered during the latter stages, thereby potentially affecting the physiological response to a peak period sequence [16, 17, 23]. It is therefore of interest to compare the physiological underpinnings and the ability to maintain performance during peak periods occurring at different stages throughout a game.

The aim of the present study was therefore to examine the muscle physiological responses to a novel 5‐min simulated peak‐intensity period protocol, and to compare the responses between peak periods occurring early and late in a simulated game. We hypothesized that a peak period would induce marked muscle metabolic perturbations and fatigue responses, in association with a substantial anaerobic energy system contribution. Furthermore, we hypothesized that differences would be present between early and late peak periods, with a shift away from glycolysis toward the end of the game and with a more pronounced fatigue response.

## 2 Methods

### 2.1 Ethical Approval

The project was approved by The Swedish ethics review authority (dnr 2021‐05974‐01) and was conducted in correspondence with the standards of the Declaration of Helsinki except for registration in a database. All participants were fully informed of any potential risks and discomforts associated with the study and provided their written informed consent to participate.

### 2.2 Participants

Eleven well‐trained male soccer players volunteered to participate in the study (mean ± SD); age: 22 ± 3 years; weight: 79.7 ± 7.0 kg; stature: 185 ± 6 cm and Yo‐Yo IR2 performance: 1109 ± 74 m. The fiber type distribution of the participants was 44 ± 8% type I fibers, 53 ± 7% type IIa fibers, and 3 ± 2% type IIx fibers. The participants were all competing in the second division in Sweden and engaged in regular soccer training and strength training sessions 4–5 and 1–2 times weekly, respectively. The study was conducted during the end of the pre‐season with games (competitive or friendly) approximately once a week. The inclusion criteria were a Yo‐Yo IR2 performance above 1000 m to ensure the inclusion of well‐trained players only, reflecting the high‐intensity exercise capacity of elite soccer players [24]. This test was performed within 2 weeks prior to the study and as previously described [24].

### 2.3 Experimental Design

**FIGURE 1:**
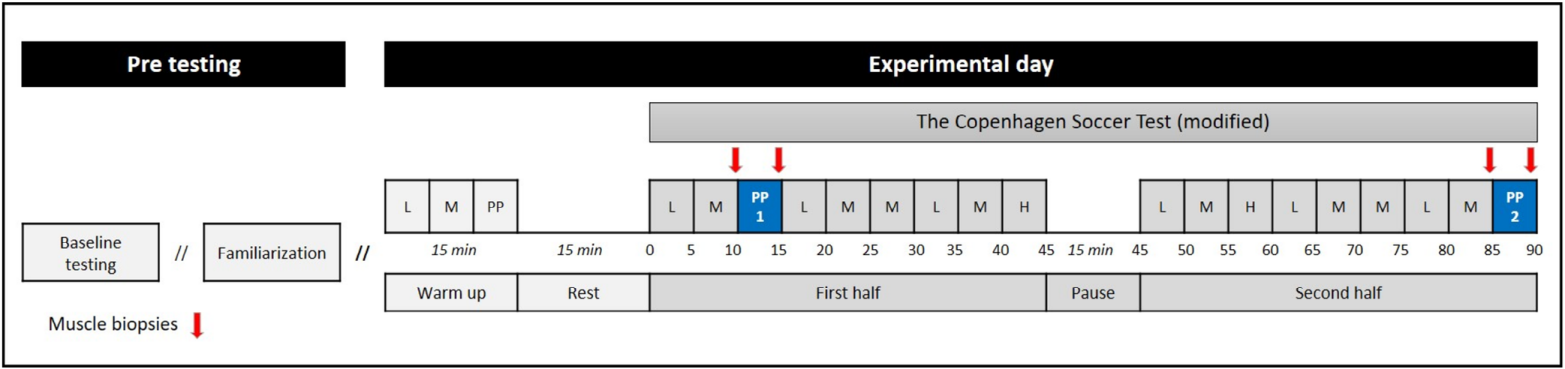
Study overview of pre‐testing and experimental day timeline. H, high‐intensity block; L, low‐intensity block; M, medium‐intensity block; PP, peak period block.

The participants completed a simulated soccer game (The CST) consisting of repeated ~5‐min intervals of football‐specific running and ball‐handling activities for 2 × 45 min interspersed with a ~15‐min half‐time intermission. The test has been described in detail elsewhere [19], but is designed to reflect the physiological demands of a competitive soccer game with similar heart rate and muscle metabolite responses, including muscle glycogen degradation rate. Contrasting a regular soccer game, this results in a standardized activity pattern throughout the game and with the ability to incorporate frequent performance measures. The 5‐min blocks differ in the amount of high‐intensity running distance and are categorized accordingly as low‐, medium‐, or high‐intensity periods; see Figure 1 for distribution throughout the match simulation. As a unique feature of the present study, two of the high‐intensity 5‐min intervals of the CST were substituted with two standardized 5‐min “peak”‐intensity periods created based on match analyses of 39 players across 45 games from the highest Swedish national level [25]. Accordingly, the 5‐min period with the highest amount of activity during each game was categorized as the peak‐intensity period based on analyses of a composite of various movement categories using a moving average and formed the basis of the conception of the 5‐min peak simulation. The 5‐min peaks were placed in the beginning (from minute 10–15) and end (from minute 85–90) of the game to compare the physiological and performance responses in peak periods at vastly different game stages. The first peak 5‐min period was placed ~10 min into the game to allow for full activation of the aerobic energy system and increases in muscle temperature [26]. Both peak periods were preceded by a low and medium‐intense block to ensure the same instantly prior activity.

The players were familiarized with the CST protocol in the week before the experimental day by completing ~15 min of the protocol (low, medium, and high‐intensity 5‐min intervals), as well as the peak 5‐min simulation period. On the experimental day, muscle biopsies were obtained in association with the peak periods as described in more detail below. Furthermore, a local positioning system (LPS tracking; KINEXON Precision Technologies, KINEXON ONE, version 1.0, Munich, Germany) was installed in the indoor soccer field to capture the activity pattern of the players during the peak periods specifically, while heart rate was monitored continuously (Polar Team Pro, Polar Electro Oy, Kempele, Finland). The players were encouraged to adhere to their habitual dietary intake prior to the experimental game and were allowed to drink 1 L of water during the match simulation, but no other ergogenic aids. Accordingly, no intake of caffeine was allowed for at least 12 h prior to the game simulation, while the players refrained from strenuous physical activities for at least ~48 h prior to the experimental day. The experimental procedures were performed in an indoor hall with fourth generation artificial turf allowing for standardized and thermoneutral conditions (~18°C).

### 2.4 Experimental Procedure

On the experimental day, the players arrived at the indoor arena between 9 am and 1 pm. The participants were encouraged to eat at least one carbohydrate‐rich meal before arriving at the arena. The players initiated the experimental procedures by performing a ~15‐min warm‐up period consisting of a low‐intensity and a medium‐intensity 5‐min round followed by ~3 min of the peak‐intensity period simulation performed at increasing exercise intensity each minute to allow for further familiarization and warm‐up prior to the actual test procedures. Following the warm‐up, the players rested in the supine position on a portable bed for ~15 min while preparations were made for the first biopsies to be obtained in association with the first peak period. This included the application of local anesthetics (5–10 mL 1% lidocaine) to allow for small incisions made through the skin, which was subsequently covered with sterile strips and a band‐aid. After these procedures, the CST was initiated with performance measures incorporated each 5‐min round including a 20‐m shuttle sprint at the end of each round and ratings of perceived exertion collected using the Borg CR10 scale. Instantly before and after the 5‐min peak periods, muscle biopsies were obtained (100–200 mg w.w. per sample) from m. *vastus lateralis* using the Bergström needle technique with suction. Also, after sterilization, blood was sampled from an antecubital vein at the same time points. When muscle biopsies and blood samples were obtained before each peak period, the players rested completely for 1 min to ensure standardized sampling conditions and a partial normalization of muscle metabolite homeostasis. After each peak period, the players finalized their last running bout right next to the portable bed, enabling fast sampling of the post‐exercise muscle biopsy (within ~10–20 s), which was instantly frozen in liquid nitrogen. During the ~15‐min halftime intermission, preparations were made for the biopsies to be obtained in association with the second peak period, which was completed in accordance with the first peak period procedures.

### 2.5 The Peak Period Simulation

The peak period simulation was designed according to the 5 min most demanding passage of soccer match play identified based on a composite kinematic activity criterion variable [25, 27]. The peak period simulation performance variable was composed of the total distances covered concurrently across moderate‐speed running (MSR; 15.0–19.8 km h^−1^), high‐speed running (HSR; 19.8–25.5 km h^−1^), and sprinting (SPR; > 25.2 km h^−1^) velocity thresholds. The peak period simulation consisted of five repetitions of 1 min of intermittent exercise including soccer‐specific movements. The velocity of the player throughout the drill was established based on the time required to cover specific distances. The running performance of the players across the peak period simulation was similar to that observed during match play [25, 27]. All players were familiarized with the protocol, initially by a walk‐through with verbal instructions provided by the researchers and subsequently in a timed manner, in the week prior to the experimental day. Players were verbally coached and encouraged throughout the completion of the drill. All testing sessions were conducted indoors on artificial turf. In brief, the test consisted of the following:

From a standing start, players ran forward before decelerating to a full stop at the 15‐m mark and waiting for the next action (5 s duration in total). Players then completed ~43 m in ~15 s, including 13 m of linear running, a “figure 8” slalom running pattern, and 2.5 m of two high‐intensity accelerations interspersed by a backward jog. Players arrived at a timing gate at a complete stop, lasting 2 s, and then performed the first measured maximal sprint over 15 m (measuring the time across the 10‐m sprint distance) before returning to the start gate by covering 28 m in 22 s. From here, the players performed a 13 m (in 3 s) diagonal run to a complete stop, lasting 2 s. Players then performed the second measured maximal sprint over 15 m (measuring sprint time across 10 m) and returned to the starting spot by completing a 28 m run in 8 s. Accordingly, maximal sprints were incorporated in the early and late parts of each of the five repetitions of the peak period protocol to assess performance changes.

### 2.6 Muscle Analyses

The muscle biopsies were stored at −80°C following the experimental day until further processing. Muscle fiber‐type distribution was determined from homogenate using gel electrophoresis and quantified densitometrically as previously described [10]. Freeze‐dried muscle (~1 mg) and sample buffer (~250 μL, 10% glycerol, 5% 2‐mercaptoethanol, 2.3% SDS, 62.5 mM Tris, 0.2% bromophenolblue at pH 6.8) were mixed together and boiled for 3 min in water before loading on an SDS‐PAGE gel (6% polyacrylamide (100:1 acrylmid:bis‐acrylmid), 30% glycerol, 67.5 mM Trisbase, 0.4% SDS, and 0.1 M glycine) with three different protein quantities (8 μL, 12 μL, and 15 μL corresponding to 0.03, 0.045, and 0.06 mg protein). The gels were run for a minimum of 42 h at 4°C and 80 V, and Coomassie staining was utilized to visualize the MHC bands. To determine the muscle fiber composition, an average from two muscle biopsies was used from each participant from three different lanes for each biopsy.

Whole‐muscle glycogen content was measured spectrophotometrically (Beckman DU 650; Beckman Instruments, Fullerton, CA). In brief, ~1.5–2 mg dw freeze‐dried muscle tissue was boiled in 1 M HCL and the supernatant mixed with 1 mL of reagent solution (1 M Tris buffer, distilled water, 100 mM ATP, 1 M MgCl_2_, 100 mM NADP^+^ and glucose‐6‐phosphate dehydrogenase). The reaction was initiated with the addition of hexokinase and the absorbance recorded before and after 60 min of reaction to calculate the muscle glycogen content.

Muscle metabolites (lactate, ATP, PCr, and Cr) were measured using freeze‐dried muscle tissue (~10 mg dw) extracted with 0.5 m HClO_4_ by the use of enzymatic methods [10]. To account for weighing variability and the presence of non‐muscle constituents, all results were normalized to total protein content (PCr + Cr). This was done by dividing the muscle metabolite values of the individual subject by the individual total PCr + Cr level and multiplying by the average of the whole sample (124.2 mmol kg^−1^ dw).

### 2.7 Blood Analyses

The analyses of lactate, glucose, and creatine kinase were performed at the Clinical Chemistry Department of Sahlgrenska University Hospital using the Alinity C analyzer (Abbott Laboratories Chicago, IL, USA) and standardized enzymatic methods.

### 2.8 Performance Testing and RPE Scores During the CST

A 20‐m shuttle sprint test was incorporated in each CST round and timed using photocells (Witty Gate Wireless Training Timer Photocells, Microgate, Italy, with a precision of 0.001 s). The test was placed ~4.5 min into the round and initiated after a countdown with the player positioned standing with one foot on a line 50 cm before the first timing gate to avoid triggering the gates prematurely. Following a 20‐m linear sprint, the player had to decelerate and make a sharp turn with one foot touching a line before returning through the timing gates. The players were familiarized with the shuttle sprint test thrice during the familiarization visit prior to the experimental day, as well as twice during the warm‐up (at sub‐maximal speeds) for the experimental match procedure.

RPE scores were collected after each 5‐min round as the player walked toward the start position to proceed with the subsequent round. The participants were familiarized with the scale prior to the experimental day and a standardized wording used to frame the question: “How hard did you perceive the last 5‐min round”.

### 2.9 Tracking

A local positioning system (KINEXON Precision Technologies, KINEXON ONE, version 1.0, Munich, Germany) was used to track activity patterns during the two peak periods. Sixteen antennas were positioned around the field. Each player was equipped with a sensor placed in a pocket between the scapulae in a manufacturer‐designed vest. Positional data were collected at a sampling frequency of 25 Hz. Data were analyzed using the proprietary software with firmware and software versions corresponding to the latest versions at the time of data collection (March 14–16, 2022). Distance covered in total and with low speed (< 7.2 km h^−1^; LOW), moderate speed (7.2–14.4 km h^−1^; MOD), high‐speed running (14.4–19.8 km h^−1^; HSR), very high‐speed running (19.8–25.2 km h^−1^) and sprinting (> 25.2 km h^−1^) were extracted from the data. Moreover, maximal running speed and number of accelerations > 3 m s^−2^ and decelerations < −3 m s^−2^ were collected. Due to technical challenges, tracking data were only captured for 8 of the 11 participants during the match and peak period simulations.

### 2.10 Statistical Analyses

Data normality was assessed using Q–Q plots and histogram models. Two‐way ANOVA with repeated measures were used to evaluate differences in muscle metabolite levels, blood values, and sprint performance in peak period 1 vs. 2. An additional paired *t*‐test was performed to specifically assess potential differences in fastest sprint time across peak periods. One‐way ANOVA with repeated measures was used to evaluate changes in RPE, 20‐m shuttle sprint times, and heart rate during each 5‐min bout of the CST. In this regard, RPE data was treated as quasi‐linear since the current version of the Borg Scale (CR10) was created to reflect exertion in even increments. To confirm these RPE findings, a Wilcoxon Signed Rank test was applied for the primary comparison of PP1 vs. PP2. Otherwise, Holm–Sidak post hoc tests were applied when significant interactions were detected. Paired *t*‐tests were applied to compare heart rate and running distances in peak 1 vs. peak 2. The significance level was set at *p* ≤ 0.05, and data were presented as mean ± SD.

## 3 Results

### 3.1 Muscle Metabolic Responses During Peak Periods 1 and 2

**FIGURE 2:**
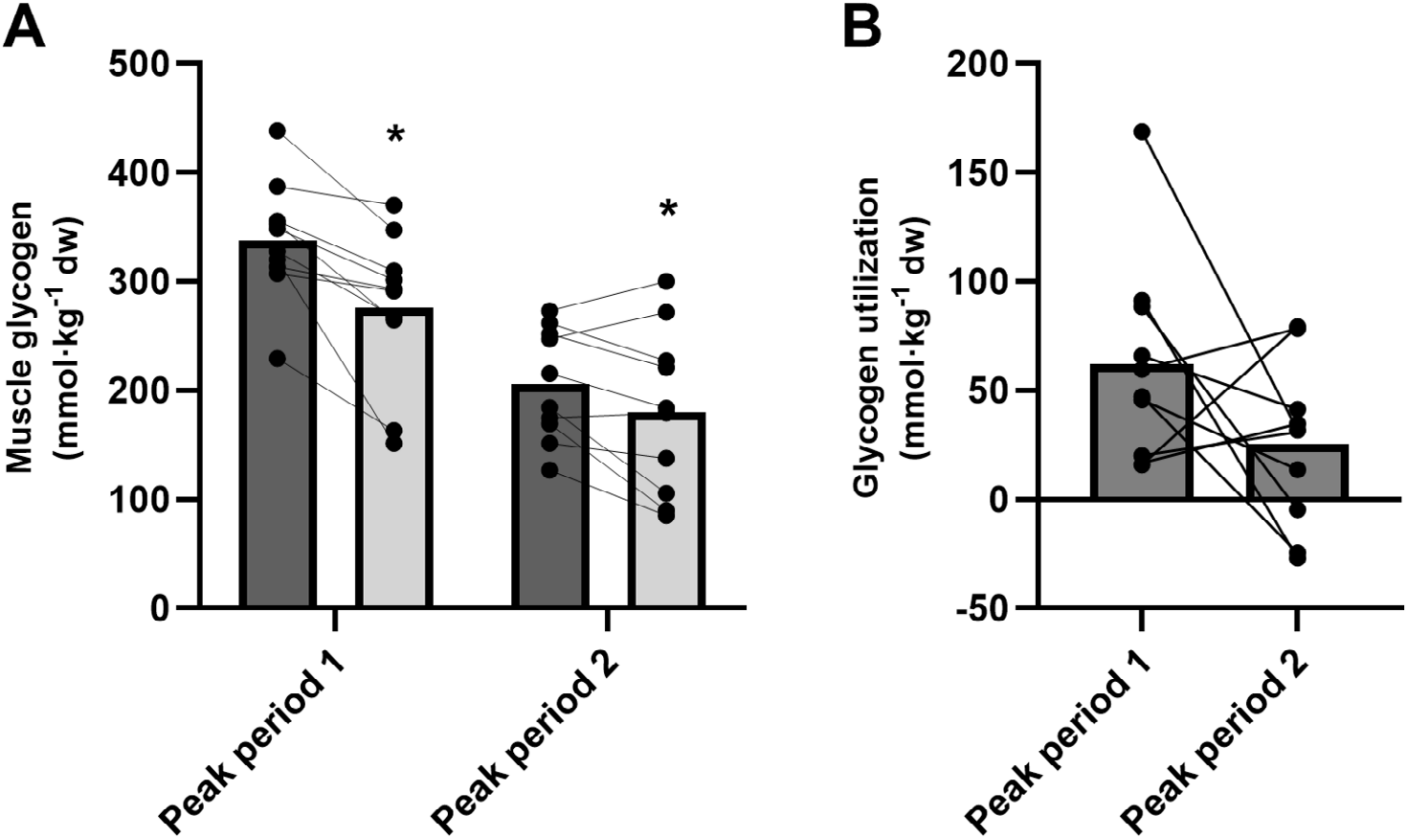
Muscle glycogen metabolism including (A) muscle glycogen levels before (dark gray) and after (light gray) peak period 1 and peak period 2 and (B) muscle glycogen utilization during peak period 1 and peak period 2. Data are presented as means and individual values (*n* = 10). *Significant difference compared to pre of the respective peak period (main effects, *p* ≤ 0.05).

Main effects of time (*p* < 0.001) and peak period (*p* < 0.001) were present, showing that muscle glycogen concentrations decreased during the peak periods, and that the values were overall lower during PP2. However, the interaction effect between time and peak period did not reach statistical significance (*p* = 0.115), indicating that the change in glycogen from pre to post was not different between the two peak periods despite differences in absolute glycogen utilization levels (decrease in PP1 by 62 ± 46 mmol kg^−1^ dw from 338 ± 55 to 276 ± 70 and by 25 ± 37 mmol kg^−1^ dw in PP2 from 206 ± 51 to 180 ± 75, see Figure 2A,B). Additionally, baseline levels were significantly lower prior to PP2 compared to PP1 (*p* < 0.001), reflecting the decrease in muscle glycogen levels across the match simulation.

**FIGURE 3:**
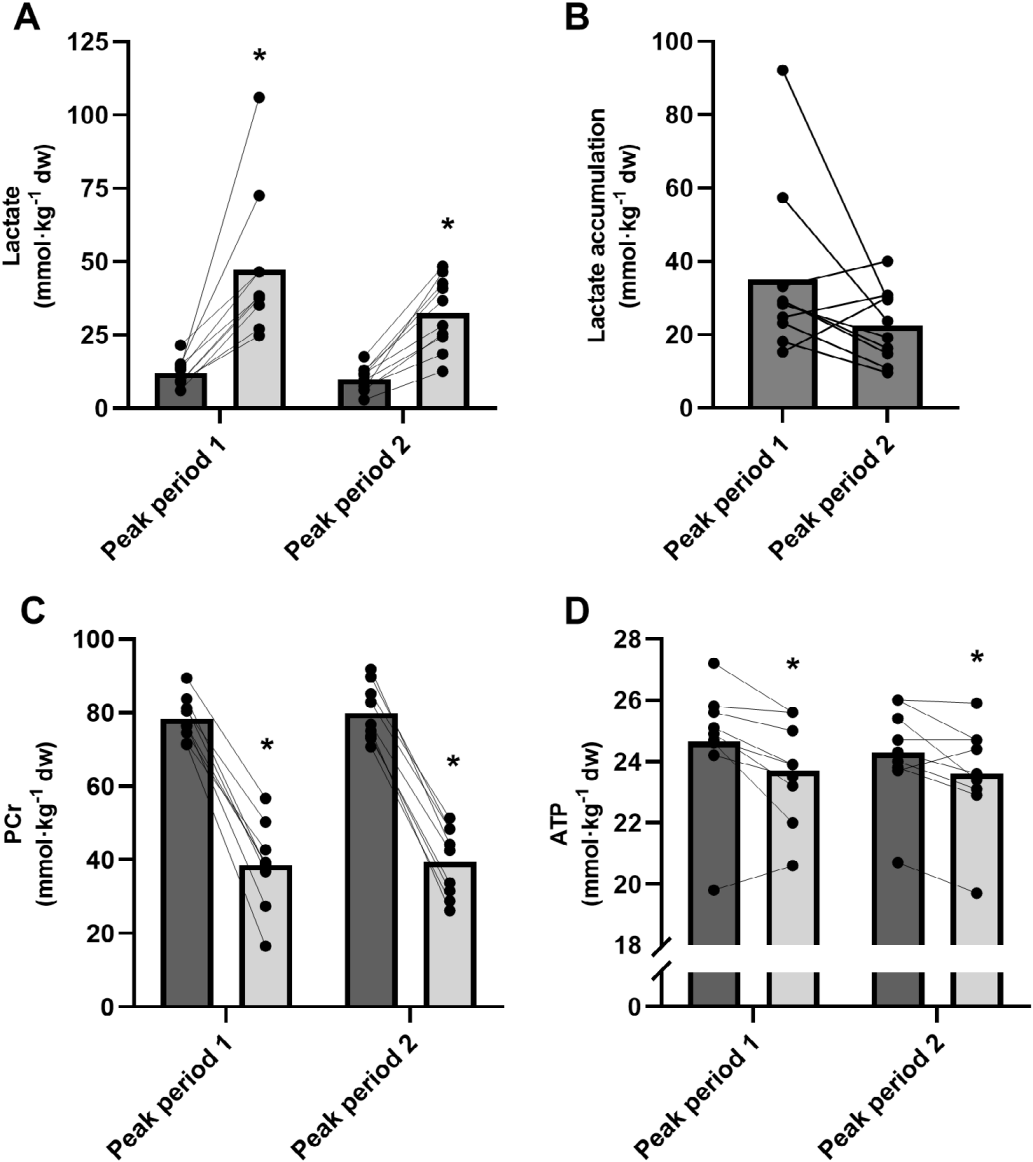
Muscle metabolites before (dark gray) and after (light gray) peak period 1 and peak period 2 including (A) muscle lactate, (B) lactate accumulation within each peak period, (C) phosphocreatine (PCr) and (D) ATP. Data are presented as means and individual values (*n* = 10). *Significant difference compared to pre of the respective peak period (main effects, *p* ≤ 0.05).

Main effects of time (*p* < 0.001) but not peak period (*p* = 0.051) were present for muscle lactate concentrations, which increased ~4 fold after the peak periods, with no significant interaction effect (*p* = 0.108), see Figure 3A,B. Phosphocreatine levels were lowered by ~50% following both peak periods (main effect; *p* < 0.001) with no effect of peak period (*p* = 0.412) and no interaction effect (*p* = 0.877), Figure 3C. The muscle ATP concentration decreased 4% during the peak periods (main effect; *p* = 0.004) with no effect of peak period (*p* = 0.932) and no interaction effect (*p *= 0.241), Figure 3D.

### 3.2 Blood Responses

Plasma glucose concentrations increased more (interaction effect; *p* = 0.020) during PP1 (5.2 ± 0.5 mmol L^−1^ to 6.1 ± 0.9 mmol L^−1^, *p* < 0.001) compared to PP2, where no change was evident (5.3 ± 0.5 mmol L^−1^ to 5.4 ± 0.7 mmol L^−1^, *p* = 0.437). Plasma lactate levels similarly increased more (interaction effect; *p* = 0.031) during PP1 (5.5 ± 2.4 mmol L^−1^ to 13.9 ± 3.6 mmol L^−1^, *p* < 0.001) than in PP2 (4.8 ± 1.6 mmol L^−1^ to 9.8 ± 2.3 mmol L^−1^, *p* < 0.001) reaching higher levels post PP1 (*p* = 0.003), with no difference before the peak periods (*p* = 0.436). Serum creatine kinase values increased more during PP2 than in PP1 (interaction effect; *p* = 0.036) from 8.2 ± 4.2 μkat L^−1^ to 8.8 ± 4.3 μkat L^−1^, *p* = 0.002 and 12.4 ± 6.3 μkat·L^−1^ to 13.3 ± 6.2 μkat·L^−1^, *p* < 0.001, respectively, with baseline values already greater before PP2 (*p* < 0.001).

### 3.3 Performance and Fatigue During Peak Periods 1 and 2

No differences in the activity pattern were present during the two standardized peak periods (see Table 1), except a 2% reduced peak sprint velocity during PP2 (*p* = 0.047).

**TABLE 1:** Locomotor activities in peak period 1 versus peak period 2.

	Peak period 1	Peak period 2	*p*
Total distance (m)	832 ± 12	829 ± 12	0.533
Max speed (km h^−1^)	23.3 ± 0.5	22.9 ± 0.5^a^	0.047^a^
Sprinting (> 25 km h^−1^) (m)	0 ± 0	0 ± 0	N/A
VHSR (19.8–25 km h^−1^) (m)	64 ± 16	53 ± 19	0.117
HSR (14.4–19.8 km h^−1^) (m)	146 ± 21	155 ± 22	0.376
MOD (7.2–14.4 km h^−1^ (m)	563 ± 26	564 ± 32	0.929
LOW (< 7.2 km h^−1^) (m)	58 ± 10	56 ± 8	0.681
Accelerations (> 2 m s^−2^) (*n*)	16 ± 2	14 ± 4	0.197
Decelerations (< −2 m s^−2^) (*n*)	15 ± 3	12 ± 3	0.085

*Note:* Data are presented as means ± SD (*n* = 8).

^a^ Significant difference between peak periods (*p* ≤ 0.05).

**FIGURE 4:**
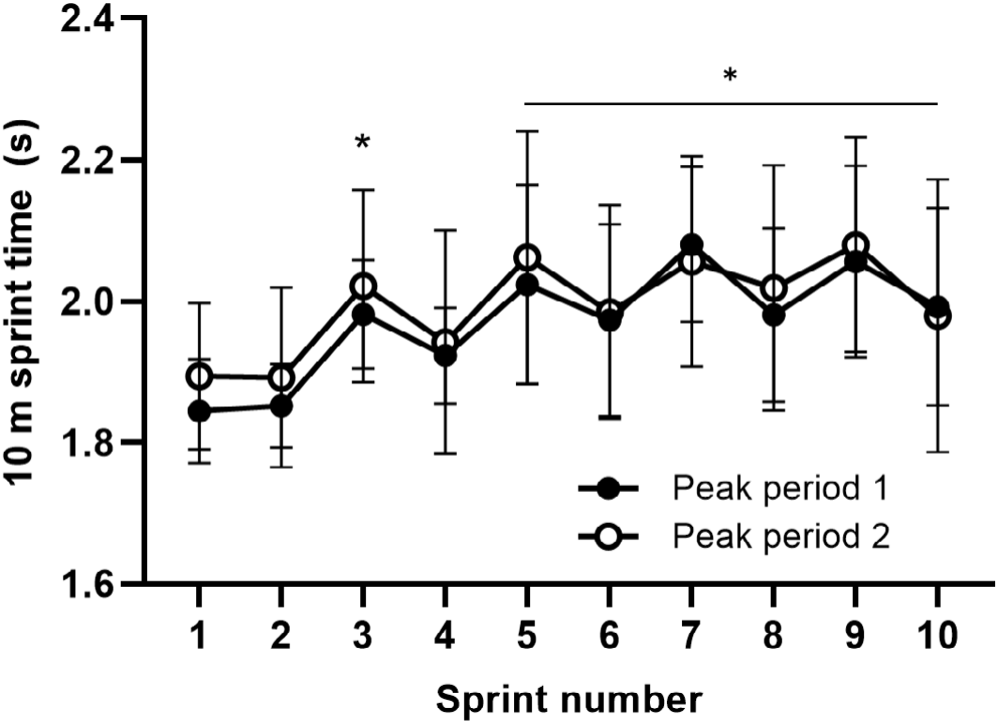
10‐m sprint performance during peak periods 1 and 2, respectively. Data are presented as means ± SD (*n* = 11). *Significant differences (main effects) compared to the first sprint (*p* ≤ 0.05).

**FIGURE 5:**
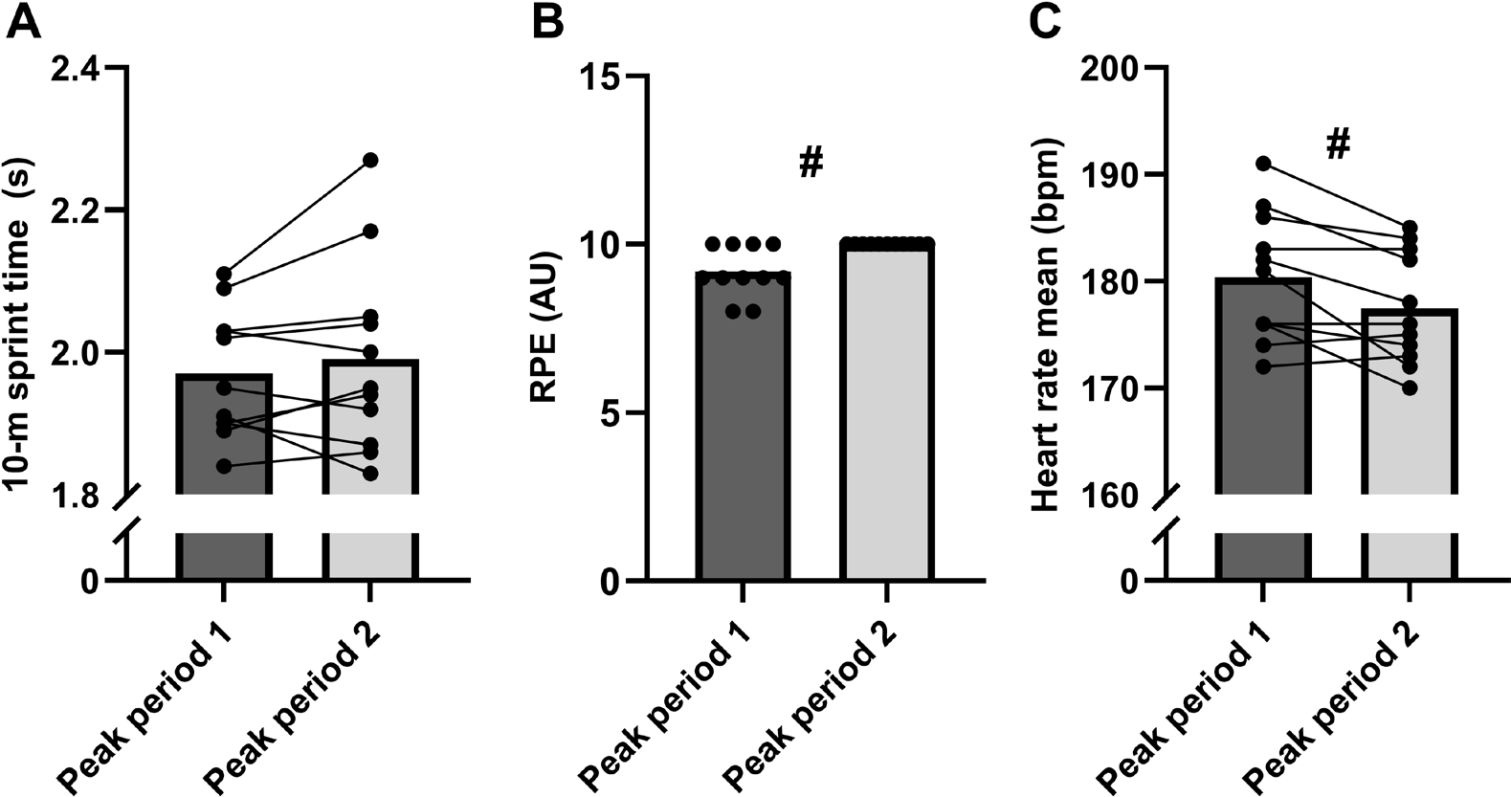
Performance and physiological responses during peak periods 1 versus 2 including (A) mean 10‐m sprint times, (B) ratings of perceived exertion (RPE) and (C) mean heart rates. Data are presented as means and individual values (*n* = 11). #Significant difference between peak periods (*p* ≤ 0.05).

No interaction effects were present for 10‐m sprint performance (*p* = 0.643), which declined by ~10% in PP1 and PP2 (main effect; *p* < 0.001) and with no difference in average performance (main effect; *p* = 0.280) or in fastest sprint time between peak periods (*p* = 0.210), see Figure 4 and Figure 5A. However, ratings of perceived exertion during the peak periods were significantly higher during PP2 (*p* = 0.023, Wilcoxon signed ranked test), increasing from 9.2 ± 0.8 to 10.0 ± 0.0 AU (all reporting maximal exertion after PP2). This occurred despite a lower mean heart rate (177 ± 5 vs. 180 ± 6 bpm, *p* = 0.016) and peak heart rate (187 ± 6 vs. 190 ± 5 bpm, *p* = 0.028) during PP2 compared to PP1 (Figure 5B,C).

### 3.4 Overall CST Responses

**FIGURE 6:**
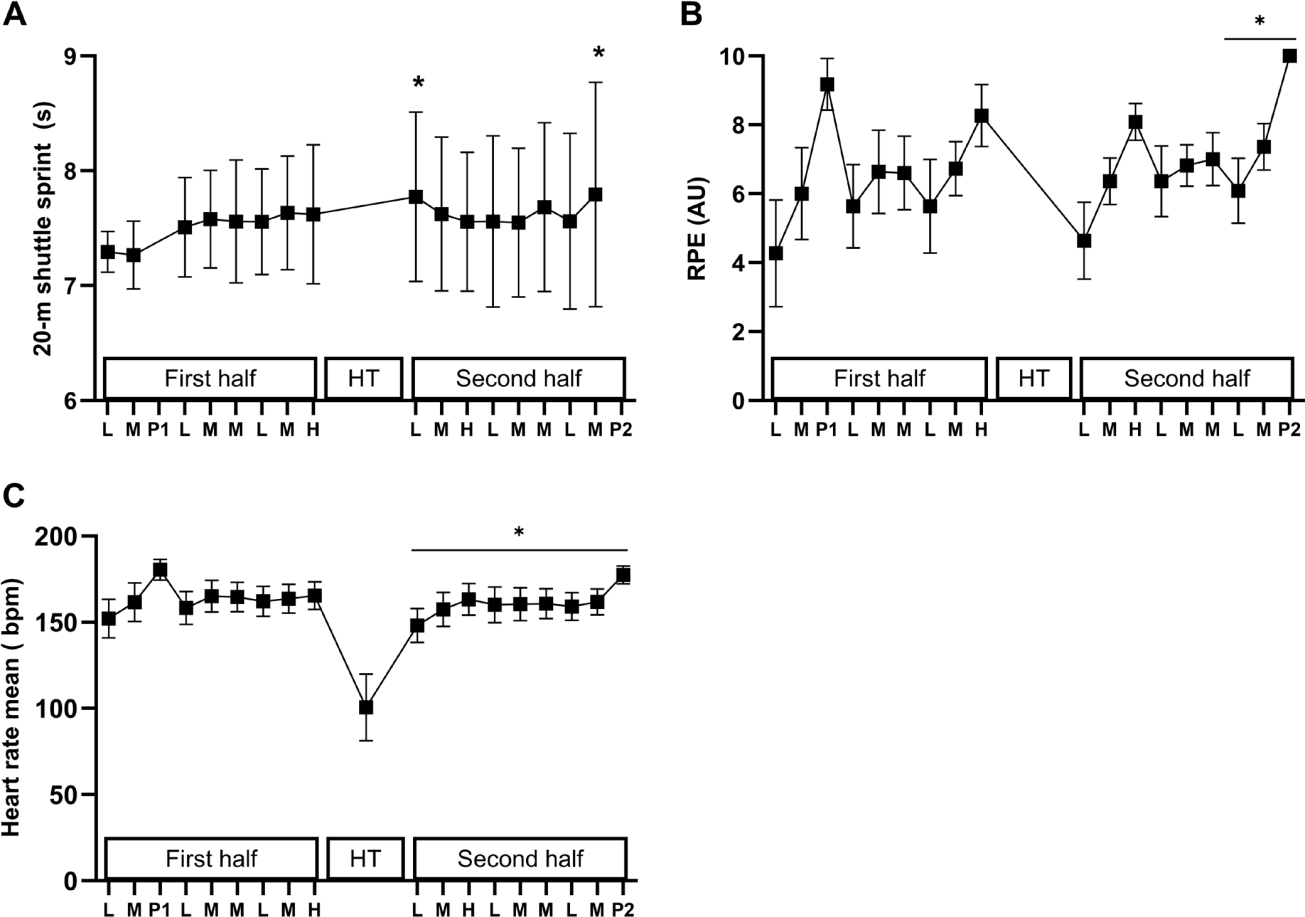
Overall responses throughout the modified Copenhagen Soccer Test including (A) 20‐m shuttle sprint performance, (B) rating of perceived exertion (RPE) and (C) heart rate. Data are presented as means ± SD (*n* = 11). *Significant difference compared to baseline for 20‐m shuttle sprint performance and significant difference compared to same time point during first half (*p* ≤ 0.05).

The sprint times during the 20‐m shuttle sprints incorporated each round of the CST were ~7% slower during the first 5‐min period following the half‐time intermission (*p* < 0.019), as well as in the final regular CST 5‐min round compared to the shuttle sprint performances during periods 1 and 2 of the game simulation (*p* < 0.011, Figure 6A). Ratings of perceived exertion increased during the CST in relation to the intensity of the round (low, medium, high‐intensity or peak period; *p* < 0.001, see Figure 6B). Moreover, an increase was evident during the latter stages of the game, with the last three rounds, including the low, medium, and final peak‐intensity round, being of higher perceived exertion than the corresponding periods during the initial 15 min of the game (*p* < 0.001). Finally, the initial low‐intensity round of each half was significantly lower in perception of effort than all subsequent bouts (*p* < 0.01). Heart rate was slightly higher during the first half compared to the second half (164 ± 9 vs. 161 ± 8 beats per minute, *p* = 0.020, Figure 6C).

## 4 Discussion

We adopted a novel simulated peak period test to examine muscle metabolism and fatigue indicators in peak‐intensity periods occurring early and late in a simulated soccer game in well‐trained male players. The major findings were that a single peak period occurring early in a game induced a large ~20% decrease in the muscle glycogen stores accompanied by high muscle lactate levels rising to ~50 mmol·kg^−1^ dw and by substantially (~50%) reduced muscle PCr levels. This demonstrates a high anaerobic energy turnover in addition to a high aerobic loading as suggested by the elevated heart rate recordings, leading to a large temporary ~10% sprint performance decline. Unclear results were present for muscle glycogen metabolism when performing a second peak period toward the end of the game, as indications of a numerically (*p* = 0.115) reduced glycogenolytic rate (~60% less glycogen degraded) were present in line with our hypothesis. This was coupled with a trend toward less muscle lactate accumulation (~40% less, *p* = 0.108) and lower blood lactate increases (40% lower, *p* = 0.031), despite an equal amount of work performed. Together, these findings offer some support for an attenuated glycolytic response during the late‐game peak period, although the evidence remains equivocal. This was accompanied by an exacerbated perception of effort during the second peak period but no aggravated decrements in 10‐m sprint performance, contrasting our initial hypothesis.

The acute metabolic perturbations following the peak period simulation are of a larger degree than those previously reported in sub‐elite male players [16] and in elite female players [17] following more randomly selected intense in‐game sequences and with a longer biopsy sampling delay. Indeed, we reported ~50% reductions in muscle PCr concentrations down to a mean of ~40 mmol·kg^−1^ dw, which is similar to or slightly higher levels than those observed after a maximal 20‐s sprint effort [15, 28] or during laboratory‐based repeated sprint activities [11], highlighting the intense peak period activity nature. Notably, these reductions in PCr are markedly greater than outcomes observed after ice hockey gameplay, despite the intense activity pattern inherent in hockey, which may in part relate to time differences in the biopsy extraction and/or the more prolonged duration of peak period exercise in soccer [29]. Importantly, while the post peak period PCr levels were not fully depleted, actual post‐exercise levels instantly after cessation of exercise may have been close to exhaustion considering the fast initial PCr resynthesis of 1–3 mmol·kg^−1^ dw·s^−1^ [11] in relation to our ~15 s sampling delay. In particular, critically low levels may have been present in a proportion of individual fibers considering the large heterogeneity in PCr concentrations at the single‐fiber level [30]. Hence, the reductions in PCr and concomitant increases in inorganic phosphate (although not measured) are likely candidates to suppress exercise tolerance in peak period scenarios [31]. In addition, the subsequent PCr restoration will be incomplete for a significant duration due to the time needed for full restoration after severe depletion, which can last up to 3–4 min [11], with a delayed restoration particularly in type 2 fibers [32]. Thus, this aligns with performance decrements observed in the minutes following peak period game exposures in elite players [2, 7].

In line with these results, muscle lactate levels rose to ~50 mmol·kg^−1^ dw, further highlighting the elevated anaerobic energy turnover during such exercise sequences. Coupled with the high mean and peak heart rates, this suggests a highly intertwined and elevated aerobic and anaerobic energy system contribution during peak period activity. Accordingly, the glycogen breakdown rate during the first peak period amounted to ~12 mmol kg^−1^ dw·min^−1^ and resulted in the depletion of one‐fifth of the total muscle glycogen stores during only 5 min of exercise. As such, a substantial proportion of the muscle glycogen degradation may be restricted to in‐game peak period activity, as evidenced by the low subsequent decline of ~1 mmol kg^−1^ dw min^−1^ after the first peak period and until the next biopsy sampling point before peak period 2. Importantly, this estimation does not account for possible glycogen resynthesis subsequent to peak period 1 during lower intensity exercise sequences or rest intermissions facilitated by the increased lactate concentrations and glycolytic intermediate accumulation, as has been shown in other exercise modalities [33]. In addition, these peak period breakdown rates are twice as high as those observed during continuous exercise at 90% VO_2max_ due to the exponential increase in glycogen breakdown rate at supramaximal exercise intensities [21]. The glycogen utilization is, however, lower than reported during a similar exercise time of ~4 min sprint cross country skiing [34], demonstrating a 22% reduction in muscle glycogen corresponding to 34 mmol kg dw^−1^·min^−1^ as opposed to the present 12 mmol·kg^−1^ dw min. This is likely explained by a disproportionate increase in skeletal muscle glycogen breakdown with increased intensity, which seems to be the highest with high muscle glycogen contents [34]. Thus, monitoring the occurrence of peak periods during soccer matches may be important to comprehend both in‐game and end‐game fatigue responses, since glycogen reductions may be a major determinant of end‐game exercise tolerance, in particular due to single fiber and compartment‐specific depletion [10, 20]. However, contrary to our hypothesis, no major exacerbated performance deteriorations were observed during peak period 2 in the present study, as discussed in a subsequent section.

During peak period 2, a trend was apparent toward a reduced glycogenolytic/glycolytic energy contribution through the lower absolute glycogen degradation levels and muscle lactate accumulation coupled with an attenuated rise in blood lactate levels. However, the declines in PCr and ATP were similar, suggestive of an unaltered phosphagen metabolism. These results confer with repeated sprint studies showing unaltered PCr metabolism during short‐term repeated sprint exercise, provided that sufficient recovery is enabled to maintain adequate levels [8, 15]. On the contrary, the glycogenolytic rate has consistently been shown to be downregulated acutely during repeated intense exercise, possibly through inhibition of glycolytic enzymes via glycolytic intermediate or H^+^ accumulation and/or as a result of a decreased exercise tolerance, and partly compensated for by an increased oxidative phosphorylation [8, 9]. However, the present study is, to the best of our knowledge, the first to assess the metabolic responses to intense intermittent exercise occurring early or late in a complex exercise session (soccer match simulation). Potential explanations for the possibly altered muscle physiological response could relate to the lowered muscle glycogen levels, as pre‐exercise glycogen concentrations consistently have been shown to affect the degradation rate during prolonged exercise [35, 36]. Notwithstanding, this has not been a common finding during supramaximal exercise where perturbations in intracellular homeostasis seem to potently trigger maximal rates of glycogenolysis irrespective of initial glycogen level, at least when glycogen concentrations are not severely lowered [21]. Despite this, the results are in line with reductions in glycogen breakdown rates observed during standardized high‐intensity intermittent cycling exercise employed for an extended duration [10]. This may associate to either a very marked decrease in muscle glycogen content and/or a shift toward higher reliance on FFA and/or blood glucose oxidation, which typically increase with extended exercise duration, and possibly an increased total aerobic energy system contribution [37]. These findings are supported by several studies showing an increase in plasma FFA and glycerol at the end of soccer games indicative of a higher fatty acid metabolism [16, 17, 23]. Moreover, as highlighted previously, maximal β‐hydroxy‐acyl‐CoA‐dehydrogenase (HAD) activity in vastus lateralis (a marker of muscle β‐oxidative capacity) was previously shown to correlate to the distance‐deficit from the first to the second half of a soccer game and to explain ~30% of the variance in high intensity running in the final 15‐min [22]. This suggests that the ability to rely more on fat oxidation and spare muscle glycogen could be an important attribute to counter end‐game fatigue responses [1].

Surprisingly, players were able to maintain their sprint performance during peak period 2, which was placed in the final 5 min of the game, with a similar ~10% decline in 10‐m sprint time from before to after the peak period sequence. Only a small (2%) reduction in peak sprint velocity was found in peak period 2 versus peak period 1, with no difference in mean 10‐m sprint ability. This was despite severe reductions in muscle glycogen concentrations to a mean of 180 mmol kg^−1^ dw after peak period 2, and in contrast to the literature where performance deteriorations are consistently observed after substantial amounts of prior exercise during both laboratory‐based intermittent exercise protocols [10], as well as soccer‐specific simulations [38] and experimental games [16, 23]. Furthermore, this contrasts the ~7% reduction in 20‐m shuttle sprint performance observed just prior to the second peak period in the present study. One explanation may be that some degree of pacing took place during the peak period sprints due to the prolonged duration and high intensity of the peak period simulation, which yielded maximal or near‐maximal ratings of perceived exertion. In support, a high number of sprints, as in the peak period simulation, has previously been shown to increase the susceptibility for pacing in repeated sprint protocols [39]. Moreover, ratings of perceived exertion did increase significantly during peak period 2, with all players reporting maximal exertion, indicative of an exacerbated fatigue response, and possibly explaining how players were able to maintain their sprint performance only through an increased effort of exercise, which is important for potential pacing strategies adopted during peak in‐game scenarios.

Since the previously mentioned initial study by Mohr et al. [2], introducing peak periods as a phenomenon causing fatigue transiently in a soccer game, several studies have been conducted over the last two decades focusing on the peak‐intensity periods [3, 6, 7]. The peak period test applied in the present study is based on a detailed analysis of 56 games to resemble a realistic worst‐case scenario of a real game [25, 27]. Thus, a strength of the present study is the application of a novel peak period test resembling peak‐intensity activity periods in competitive soccer games in combination with the CST, which has been shown to exert a comparable physiological loading as a soccer game [19]. Indeed, the peak period test was based on a composite analysis of various locomotor variables in the most demanding 5‐min periods in real competitive games (see Section 2) [25]. However, it must be acknowledged that a peak period scenario in a soccer game may differ markedly at the individual level and between games, inferring that the peak period simulation adopted herein should be considered a reflection of a realistic scenario that a player could face during a peak‐intensity sequence rather than a fixed entity. The obtainment of muscle biopsies before and after a peak period early and late in a game provides for the first time an experimental setting where muscle metabolism can be studied in detail in the initial and end phases of a game. The application of a standard peak period model and the CST aids on one hand to improve standardization of the experiment, but on the other hand may compromise the ability to translate the findings directly into real game scenarios where substantial inter‐individual and inter‐game variability is present as discussed above [18]. Moreover, since muscle metabolic and fatigue responses can differ between sexes, the present results mainly apply to male players. Finally, as the within‐subject variability in muscle glycogen degradation between peak period 1 and peak period 2 was greater than anticipated, we may have been underpowered to confidently conclude on the differences in glycogenolytic rate early and late in a game, and additional studies are warranted to substantiate these findings.

In conclusion, the simulated peak‐intensity 5‐min periods applied in the present study, which were constructed to reflect a potential worst‐case game scenario, induced large reductions in muscle glycogen concentrations, substantial lactate accumulation, and PCr degradation, demonstrative of a high anaerobic energy turnover, and were accompanied by significant fatigue development. In addition, while high‐energy phosphate metabolism remained similar during a peak period performed at the end of the game, the glycogenolytic rate appeared attenuated, accompanied by an exaggerated perceptual but similar sprint performance deterioration.

## 5 Perspective

The present work provides insights into muscle metabolism and fatigue patterns during peak‐intensity exercise periods occurring during the early and late phases of a simulated soccer game. Evidently, in the present study, the fatigue development during each peak period was substantially more pronounced than fatigue in the latter compared to the initial stages. This highlights the importance of preparing specifically for the worst‐case game demands and to ensure a highly developed ability to limit performance deteriorations during such scenarios. In this case, the peak‐intensity period simulation may provide a specific tool to assess this ability in players. However, despite no clear performance impairments in the late compared to the early peak period, it should be emphasized that perceptual responses were substantially higher and that both the potential occurrence of pacing and differences in muscle loading patterns in the simulated game scenario compared to a real competitive setting may have influenced these outcomes. Therefore, future work is needed to support our findings, e.g., by modifying the peak period sprint protocol and/or adopting the protocol before, during, and after real experimental games.

## Ethics Statement

Ethical approval and informed signed consent were obtained.

## Conflicts of Interest

The authors declare no conflicts of interest.

## Data Availability

The data that support the findings of this study are available from the corresponding author upon reasonable request.
